# Applying the six‐sigma methodology to determine the limits of quality control (QC) tests for a specific linear accelerator

**DOI:** 10.1002/acm2.14460

**Published:** 2024-07-29

**Authors:** Min Ma, Minghui Li, Ke Zhang, Pan Ma, Zhihui Hu, Hui Yan, Kuo Men, Jianrong Dai

**Affiliations:** ^1^ National Cancer Center/National Clinical Research Center for Cancer/Cancer Hospital Chinese Academy of Medical Sciences and Peking Union Medical College Beijing China

**Keywords:** limits, linear accelerator, quality control, six‐sigma methodology

## Abstract

**Purpose:**

We aimed to show the framework of the six‐sigma methodology (SSM) that can be used to determine the limits of QC tests for the linear accelerator (Linac). Limits for QC tests are individually determined using the SSM.

**Methods and Materials:**

The SSM is based on the define‐measure‐analyze‐improve‐control (DMAIC) stages to improve the process. In the “define” stage, the limits of QC tests were determined. In the “measure” stage, a retrospective collection of daily QC data using a Machine Performance Check platform was performed from January 2020 to December 2022. In the “analyze” stage, the process of determining the limits was proposed using statistical analyses and process capability indices. In the “improve” stage, the capability index was used to calculate the action limits. The tolerance limit was established using the larger one of the control limits in the individual control chart (I‐chart). In the “control” stage, daily QC data were collected prospectively from January 2023 to May 2023 to monitor the effect of action limits and tolerance limits.

**Results:**

A total of 798 sets of QC data including beam, isocenter, collimation, couch, and gantry tests were collected and analyzed. The Collimation Rotation offset test had the min‐Cp, min‐Cpk, min‐Pp, and min‐Ppk at 2.53, 1.99, 1.59, and 1.25, respectively. The Couch Rtn test had the max‐Cp, max‐Cpk, max‐Pp, and max‐Ppk at 31.5, 29.9, 23.4, and 22.2, respectively. There are three QC tests with higher action limits than the original tolerance. Some data on the I‐chart of the beam output change, isocenter KV offset, and jaw X1 exceeded the lower tolerance and action limit, which indicated that a system deviation occurred and reminded the physicist to take action to improve the process.

**Conclusions:**

The SSM is an excellent framework to use in determining the limits of QC tests. The process capability index is an important parameter that provides quantitative information on determining the limits of QC tests.

## 1 INTRODUCTION

A quality control (QC) test for the linear accelerator (Linac) aims to ensure that machine characteristics do not considerably deviate from the baseline values acquired during acceptance and commissioning.^1^ Many baseline variables of Linac are incorporated into treatment planning systems. As a result, the record of QC tests has indirect impacts on the treatment plans that are created for the patient treated based on that Linac. Therefore, deviation from the baseline values (such as Beam, couch, gantry, and MLC) may lead to patients receiving suboptimal treatment.^2^ A QC test that helps the physicist to decide whether the current state of Linac is acceptable by comparing the limits is performed.^3^ The limits contain the tolerance and action limits, which are the permissible deviations from baseline values.

With the development of the automation function technology of QC equipment like morning check equipment and automatic QC platforms, the time of the QC test is constantly shortening, and the number of QC tests are increasing. Some QC tests have gradually become daily QC tests. However, specification limits recommend by the Vendor are used for the daily QC tests because there are presently no comparable limits for these new daily QC tests in the professional guidance document; for example, AAPM TG‐142.^1^ However, there is little research to share on whether it is even appropriate. The limits given in the professional guidance document are group‐specific, which means it does not consider the operating state and working conditions of different pieces of radiotherapy equipment.

When there are no proper limits in a professional guidance document, setting limits based on the process is more needed. Statistical process control (SPC) uses mathematical statistics to detect signs of systematic errors in time^4^ and takes action to eliminate their effects. SPC has two applications: one is to analyze the stability of the process by using the control chart,^5^ to distinguish the random error and systematic error in the process by the threshold of the upper control limit (UCL) and the lower control limit (LCL), and to give early warnings about the abnormal factors in the process; the other is to use a process capability index to assess the process quality and provide a numerical measure of whether the QC process meets predetermined specification limits. In 2005, Pawlicki et al. first analyzed the QC process of radiotherapy through SPC.^6^ Then, this method was also applied to the QC of Linacs,^7^, ^8^, ^9^, ^10^ Tomotherapy,^11^, ^12^ and patient‐specific IMRT and VMAT QA.^13^, ^14^, ^15^ Furthermore, SPC characterizes the state of fluctuation inherent in the process, which is used to establish customized limits. In 2013, Sanghangthum et al. first proposed the use of SPC for establishing customized tolerance and action limits.^16^ Xiao et al. established tolerance limits for the patient‐specific VMAT QA using the heuristic control charts.^17^ Jin et al. discussed the feasibility of using SPC to set the limit of the daily QC tests.^18^ Limits were optimized through statistical analyses.

The six‐sigma methodology (SSM) provides a structured tool to assess and reduce failures in the process while aiming to meet rationally‐set targets.^19^, ^20^ These methods were originally developed for the manufacturing industry^21^ to provide near‐perfect services to large processes. Then, it has increasingly been successfully adapted to various healthcare settings.^20^, ^22^, ^23^, ^24^, ^25^ Recently, SSM was introduced in the process of radiotherapy.^23^, ^26^, ^27^ SSM contains five stages: define‐measure‐analyze‐improve‐control (DMAIC). The “define” stage defines the problem or improvement opportunity. The “measure” stage measures the current process performance. The “analyze” stage analyzes the process to determine the possible causes of the problem. The “improve” stage suggests a solution to the problem. Last, the “control” stage monitors the improvements to ensure their utility.

In this study, we aimed to show the framework of SSM that can be used to determine the limits of QC tests in Linac. The endpoint of our study is to recommend a workflow for establishing limits for unspecified QC tests; where possible, limits may be individually determined to meet the functional requirements of the machine QC in actual clinical radiotherapy.

## 2 METHODS AND MATERIALS

The SSM is an extremely effective framework for improving processes. SSM is based on the following five stages: the define stage; the measure stage; the analyze stage; the improve stage; and the control stage.

### 2.1 Define stage

The “define” stage is aimed at setting goals for the process and mapping out a strategy to deliver these requirements.^28^ In this study, the limits of QC tests, which were not recommended by the professional guidance document, were determined. Our goals were to analyze and determine limits for daily QC tests based on the process using statistical tools, which can improve the efficiency of physicists. Traditionally, the limits were defined by professional societies, guidance documents, or best‐practice documents, such as the recommended limits in the AAPM TG‐142 report.^1^ However, some QC tests were unspecified limits that were determined empirically via an analysis of available data or sometimes simply by using clinical experience. In this study, daily QC tests have belonged to unspecified limits.

### 2.2 Measure stage

**FIGURE 1:**
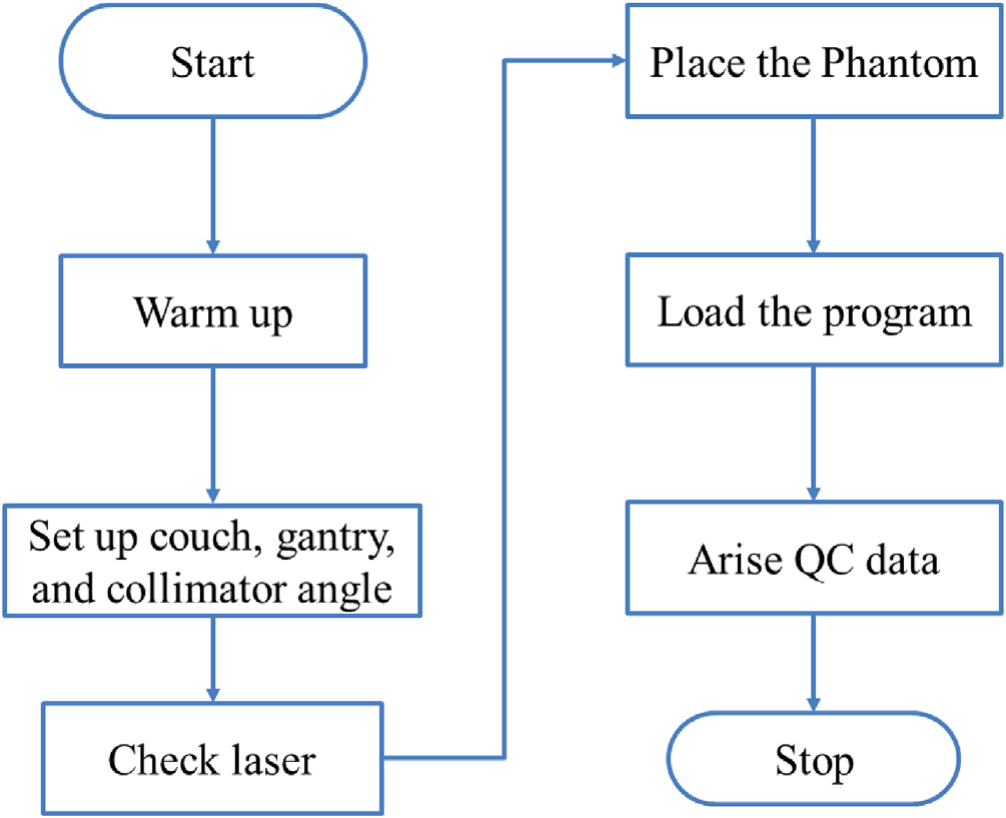
Process flow diagram of daily QC tests using the MPC platform.

The “measure” stage aims to collect reliable data on the performance of the current process.^28^ In this study, a retrospective collection of daily QC data using the Machine Performance Check (MPC, Varian Medical Systems, Palo Alto, CA, USA, Version 1.0) platform was performed from January 2020 to December 2022. The MPC is equipped for daily QC tests on the Edge Linac (Varian Medical Systems, Inc., Palo Alto, CA). The MPC is a highly‐automated, fully‐integrated KV and MV image‐based tool to examine and evaluate the machine's performance.^29^ MPC application has been evaluated as a Linac daily QC tool by some investigators.^29^, ^30^, ^31^, ^32^, ^33^ Twenty‐four QC tests for six MV x‐rays were run, including isocenter, collimation, gantry, and couch tests.^29^ Figure 1 shows the process flow diagram about daily QC tests using MPC with six steps: (1) warm up the Linac; (2) set the angle of the couch, gantry, and collimator to the zero angle; (3) check the accuracy of the laser light; (4) place the IsoCal phantom based on the laser; (5) load the automated QC program (MPC contained) on the computer; (6) generate QC data to monitor the process. In this study, the data of 24 QC tests using MPC were collected at our institution on the Varian Edge. The MPC platform automatically records data of 24 QC tests and can be exported in excel format for viewing and analysis.

### 2.3 Analyze stage

In the “analyze” stage, the determined process of the limits was proposed using statistical analyses and process capability indices.^34^ In SPC, the process capability indices are determined by assessing the performance of the under‐control process. Based on the stability process, QC data were selected to calculate the capability indices (Cp, Cpk, Cpm, Pp, and Ppk) of each QC test. The Cp characterizes the state of fluctuation inherent in the process. The Cpk considers both the fluctuation inherent in the process and the bias of mean values.^20^ The Cpm considers both deviations of target values and mean values,^35^ which is a comprehensive result of the deviation of process capability and mean values from target values.^36^ The Pp is a simple and straightforward indicator of process performance.^35^ Ppk is used to adjust the Pp for the effect of non‐centered distribution.^35^

Before the calculation, the steps of data analysis are as follows: (1) remove the data points that are beyond the tolerance (the “Tolerance (Vendor)” column of Table 3); (2) conduct Anderson‐Darling test (AD test) through Minitab19 (State College, PA, USA, Version 2020) software to determine whether the data meets the normal distribution; if it does not meet the normal distribution, carry out the Johnson transformation^37^ to convert the data to normal distribution; (3) do the individual control chart (I‐chart) of the QC data, and remove the data points that are beyond the upper control limit (UCL) and the lower control limit (LCL). The UCL and LCL is calculated using this formula^38^:

$$\;MR_{i}=\left|x_{i}-x_{i-1}\right|\;$$

$$UCL=\bar{x}+3\frac{\bar{MR}}{d_{2}}$$

$$LCL=\bar{x}\;-3\frac{\bar{MR}}{d_{2}}$$

In this study, $d_{2}=1.128$, two successive observations $x_{i-1}$ and $x_{i}$; (4) repeat the steps 2−3, and until all QC data are located within UCL and LCL. The under‐control QC data were used to calculate the capability indices, see Section 2.4.^39^ Next, the recommended limits of the daily QC tests in the vendor's report were used as the specification limits in calculating the capability indices.

### 2.4 Improve stage

The “improve” stage aims to select and target a solution that addresses the requirement found in the analysis stage.^26^ In this study, the capability index, *Cpm*, was used to calculate the action limits.^18^ This index is geared toward measuring the ability of a process around a target value (*T*), and it reflects the degree of process centering. The $C_{pm}$ is calculated using this formula^14^:

$$\;C_{pm}=\frac{\mathrm{UAL}-\mathrm{LAL}}{A\sqrt{\sigma^{2}+{\left(\bar{x}-T\right)}^{2}}}\;$$

where UAL−LAL is the width of the action limits when the target value (T) is zero, and A is used to adjust the widths of the limits. The mean ($\bar{x}$) and standard deviation (σ) are calculated from the data of daily QC tests only under control in the I‐chart. To eliminate systematic error, the target value was set to the mean of QC data, which was used to calculate the actual action limits. The calculation is performed with A being 6, 5, 4, and 3, and the calculated value of action limits covers the tolerance limit with the minimum A value.

Tolerance limits, which should serve as warning limits, serve as an indication that a process is changing and requires attention. The control limit of the I‐chart is ideal to use as the tolerance limit because it is designed to characterize process performance. In this study, the tolerance limit was established using the larger one of the UCL and the LCL in the I‐chart.

### 2.5 Control stage

The final step of the SSM was the “control” stage. This requires constant monitoring and control of the improved process using the I‐chart. In the “control” stage, a new process of determining limits based on the SSM for the daily QC tests was determined. In this stage, daily QC data were collected prospectively from January 2023 to May 2023 to mitigate the effect of action limits and tolerance limits. Under the new limits, the I‐chart monitors the trend of data changes and evaluates the ability to detect data changes in different limit ranges. Minitab19 (State College, PA, USA, Version 2020) software was used to create the I‐chart and calculate the capability indices.

## 3 RESULTS

### 3.1 Measure

A total of 798 sets of data including beam, isocenter, collimation, couch, and gantry tests were collected to analyze the limits of daily QC tests. Descriptive statistics regarding the initial collection of daily QC tests are detailed in Table 1. The distribution of the quality characteristics of 24 QC tests was non‐normal (Anderson‐Darling test, all *p* < 0.005).

**TABLE 1:** Summary of the descriptive statistics for daily QC data.

QC items	$\bar{x}$	σ	AD test	*p*	Normality
1. BeamCenterShift [mm]	0.193	0.119	17.6	<0.005	Not
2. BeamOutputChange [%]	0.197	0.765	3.25	<0.005	Not
3. BeamUniformityChange [%]	0.493	0.256	19.4	<0.005	Not
4. ISO KV [mm]	0.245	0.0630	14.3	<0.005	Not
5. ISO MV [mm]	0.251	0.0680	11.9	<0.005	Not
6. ISOCenterSize [mm]	0.301	0.0220	25.1	<0.005	Not
7. CollimationRatation offset [°]	0.107	0.105	14.6	<0.005	Not
8. JawX1 [mm]	0.141	0.116	25.4	<0.005	Not
9. JawX2 [mm]	0.0456	0.0883	27.7	<0.005	Not
10. JawY1 [mm]	−0.867	0.0942	3.19	<0.005	Not
11. JawY2 [mm]	0.521	0.0888	3.18	<0.005	Not
12. MLCMaxOffsetA [mm]	−0.583	0.0522	5.63	<0.005	Not
13. MLCMaxOffsetB [mm]	0.375	0.0771	92.8	<0.005	Not
14. MLCMeanOffsetA [mm]	−0.446	0.0445	8.88	<0.005	Not
15. MLCMeanOffsetB [mm]	0.241	0.0263	5.14	<0.005	Not
16. CouchLat [mm]	−0.273	0.0581	18.7	<0.005	Not
17. CouchLng [mm]	0.0700	0.0533	25.4	<0.005	Not
18. CouchPit [°]	−0.00277	0.00805	57.3	<0.005	Not
19. CouchRol [°]	−0.0204	0.00822	58.8	<0.005	Not
20. CouchRtn [°]	0.0206	0.00611	97.9	<0.005	Not
21. CouchVrt [mm]	0.0488	0.0448	3.78	<0.005	Not
22. RotationInducedCouchShift [mm]	0.461	0.113	39.9	<0.005	Not
23. GantryAbsolute [°]	−0.0954	0.0179	17.5	<0.005	Not
24. GantryRelative [°]	0.0613	0.0540	12.0	<0.005	Not

Abbreviation: AD test, Anderson‐Darling test.

### 3.2 Analysis

**FIGURE 2:**
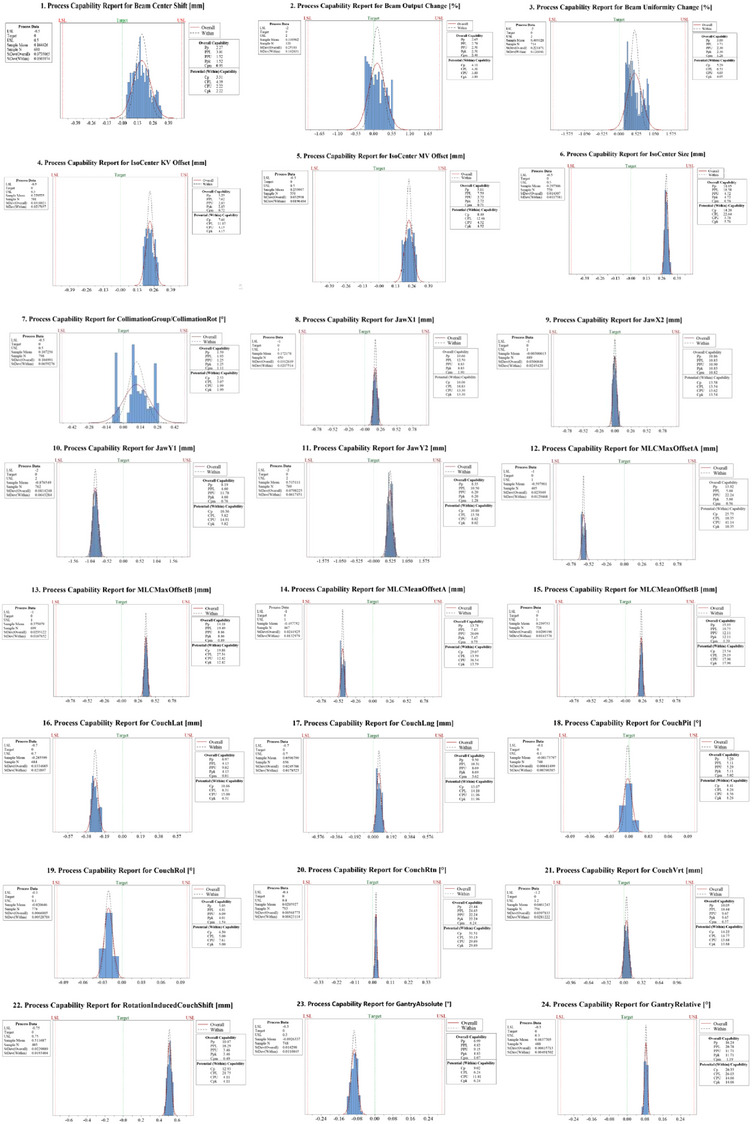
The process capability report of 24 QC tests based on the MPC platform.

All daily QC tests were used to calculate the Cp, Cpk, Cpm, Pp, and Ppk indices shown in Table 2. The data of daily QC tests under control were obtained. The Collimation Ratation offset test had the min‐Cp, min‐Cpk values, min‐Pp, and min‐Ppk values as 2.53, 1.99, 1.59, and 1.25, respectively. The Couch Rtn test had the max‐Cp, max‐Cpk, max‐Pp, and max‐Ppk values as 31.5, 29.9, 23.4, and 22.2, respectively. In Figure 2, the Collimation Ratation offset test is the farthest from the target value, and the Couch Rtn is the closest. The Cpm values of daily QC tests ranged from 0.49 to 10.82.

**TABLE 2:** Process capability indices of each daily QC test in 2020−2022.

QC tests	*N*	$\bar{x}$	σ	Cp	Cpk	Cpm	Pp	Ppk
1. BeamCenterShift [mm]	680	0.164	0.050	3.31	2.22	0.93	2.27	1.52
2. BeamOutputChange [%]	320	0.101	0.163	4.10	3.89	2.46	2.65	2.51
3. BeamUniformityChange [%]	714	0.469	0.126	5.29	4.05	1.28	3.00	2.30
4. ISO KV [mm]	581	0.229	0.022	7.66	4.15	0.72	5.23	2.83
5. ISO MV [mm]	558	0.234	0.020	8.49	4.52	0.71	5.11	2.72
6. ISOCenterSize [mm]	720	0.297	0.012	14.2	5.76	0.56	11.6	4.72
7. CollimationRatation offset [°]	798	0.107	0.066	2.53	1.99	1.11	1.59	1.25
8. JawX1 [mm]	450	0.172	0.021	16.1	13.3	1.90	10.7	8.83
9. JawX2 [mm]	489	−0.003	0.025	13.6	13.5	10.8	10.9	10.8
10. JawY1 [mm]	762	−0.872	0.054	10.4	5.82	0.76	8.19	4.60
11. JawY2 [mm]	769	0.515	0.062	10.8	8.02	1.28	8.35	6.20
12. MLCMaxOffsetA [mm]	405	−0.598	0.013	25.7	10.3	0.56	13.9	5.60
13. MLCMaxOffsetB [mm]	699	0.375	0.017	19.9	12.4	0.89	14.2	8.86
14. MLCMeanOffsetA [mm]	467	−0.458	0.013	25.0	13.6	0.73	13.8	7.47
15. MLCMeanOffsetB [mm]	728	0.240	0.014	23.5	17.9	1.39	15.9	12.1
16. CouchLat [mm]	684	−0.286	0.022	10.7	6.31	0.81	6.97	4.13
17. CouchLng [mm]	656	0.060	0.018	13.1	12.0	3.62	9.50	8.69
18. CouchPit [°]	748	−0.002	0.004	8.41	8.26	5.02	5.20	5.11
19. CouchRol [°]	774	−0.021	0.005	6.30	5.00	1.54	5.05	4.01
20. CouchRtn [°]	793	0.021	0.004	31.5	29. 9	6.24	23.4	22.2
21. CouchVrt [mm]	756	0.046	0.028	14.2	13.7	6.57	10.0	9.67
22. RotationInducedCouchShift [mm]	403	0.512	0.019	12.9	4.11	0.49	10.9	3.46
23. GantryAbsolute [°]	748	−0.093	0.011	9.02	6.24	1.07	6.99	4.83
24. GantryRelative [°]	488	0.084	0.005	20.3	14.7	1.19	16.2	11.7

*Note*: N: The number of under control points.

### 3.3 Improve

As shown in Table 3, the calculated action and tolerance limits of the same QC test were quite different from the vendor's recommended value. There are three QC tests with higher action limits than the original tolerance recommended by the vendor, namely: Iso CenterSize, MLCMaxOffsetA, and RotationInducedCouchShift. The tolerance of all QC tests is lower than the original tolerance.

**TABLE 3:** Process‐based action and tolerance limits of 24 QC tests in 2020−2022.

QC tests	Tolerance (vendor)	UCL	X	LCL	± Action limits	± Tolerance limits
1. BeamCenterShift [mm]	0.5	0.316	0.164	0.0132	0.404	0.316
2. BeamOutputChange [%]	2.0	0.588	0.101	−0.387	0.806	0.588
3. BeamUniformityChange [%]	2.0	0.847	0.469	0.0912	1.09	0.847
4. ISO KV [mm]	0.5	0.294	0.229	0.164	0.478	0.294
5. ISO MV [mm]	0.5	0.293	0.234	0.175	0.484	0.293
6. ISOCenterSize [mm]	0.5	0.332	0.297	0.262	0.547	0.332
7. CollimationRatation offset [°]	0.5	0.305	0.107	−0.0905	0.317	0.305
8. JawX1 [mm]	1.0	0.234	0.172	0.110	−0.322	0.234
9. JawX2 [mm]	1.0	0.0706	−0.00301	−0.0766	0.398	0.0766
10. JawY1 [mm]	2.0	−0.656	−0.872	−0.980	1.12	0.980
11. JawY2 [mm]	2.0	0.700	0.515	0.330	−0.813	0.700
12. MLCMaxOffsetA [mm]	1.0	−0.559	−0.598	−0.637	−1.10	0.637
13. MLCMaxOffsetB [mm]	1.0	0.425	0.375	0.325	0.876	0.425
14. MLCMeanOffsetA [mm]	1.0	−0.418	−0.458	−0.498	0.545	0.498
15. MLCMeanOffsetB [mm]	1.0	0.282	0.240	0.197	−0.428	0.282
16. CouchLat [mm]	0.7	−0.220	−0.286	−0.351	0.410	0.351
17. CouchLng [mm]	0.7	0.113	0.0597	0.00610	−0.391	0.113
18. CouchPit [°]	0.1	0.0101	−0.00174	−0.0136	0.0308	0.0136
19. CouchRol [°]	0.1	−0.00478	−0.0206	−0.0365	0.0450	0.0365
20. CouchRtn [°]	0.4	0.0333	0.0206	0.00790	0.217	0.0333
21. CouchVrt [mm]	1.2	0.130	0.0461	−0.0382	0.578	0.130
22. RotationInducedCouchShift [mm]	0.7	0.570	0.512	0.454	0.888	0.569
23. GantryAbsolute [°]	0.3	−0.0594	−0.0926	−0.126	0.157	0.126
24. GantryRelative [°]	0.3	0.0985	0.0838	0.0690	−0.116	0.0985

*Note*: X‐Target: the mean of in‐control QC data.

### 3.4 Control

**FIGURE 3:**
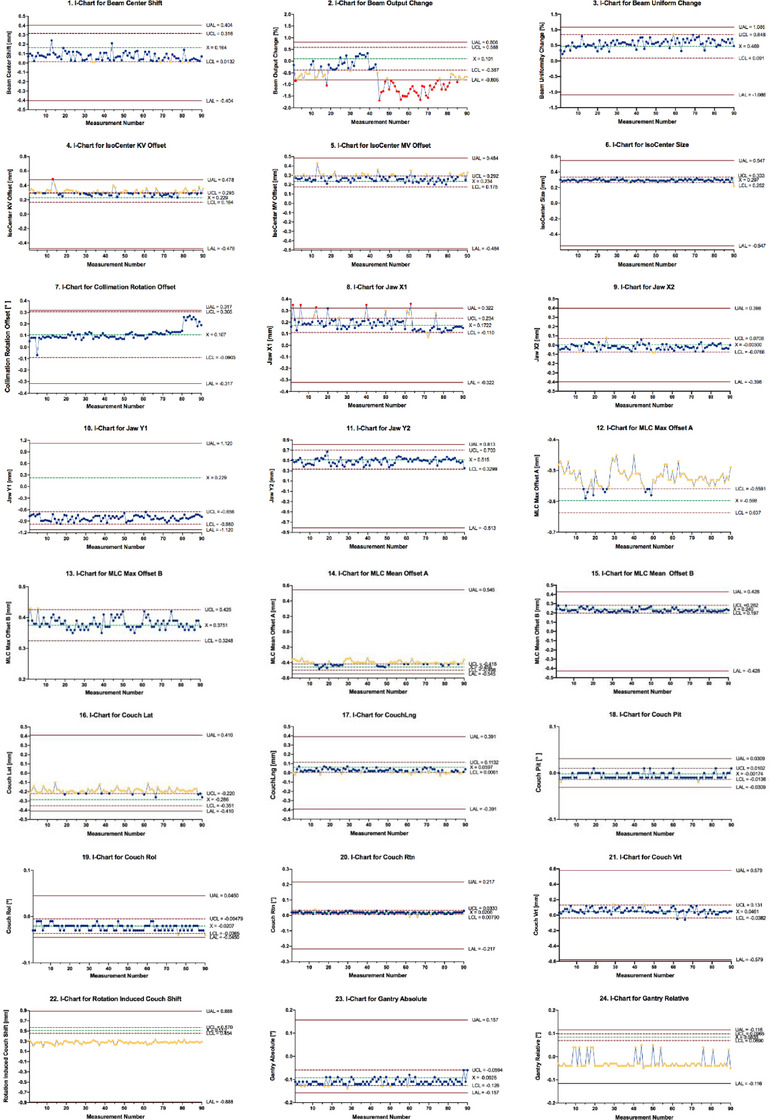
Individual control charts using the process‐based tolerance and action limit calculated using the data collected during the prospective evaluation stage. The dashed lines represent the UCLs, LCLs, and CLs of the control chart calculated from the 90 data points; the solid lines represent the range of action limits calculated from the 90 data points. The red‐filled cycles represent the data points that are beyond the tolerance and action limits. The yellow‐filled cycles represent the data points that are only beyond the tolerance limit. CL, central limit; LAL, lower action limit; LCL, lower control limit; UAL, upper action limit; UCL, upper control limit.

The I‐chart of 24 daily QC tests based on process action and tolerance limits was used. The 90 data points of each QC test were monitored and identified during the prospective evaluation stage. In the original tolerance, the data of all daily QC tests were within the tolerance range, indicating that the Linac did not fail. However, it was found in the maintenance records that the Linac did have failures. Too wide a tolerance limit did not detect the Linac failures in time, and preventive maintenance measures were taken in advance. The new limits for daily QC tests were simulated to establish priorities for improving machine QC. The I‐chart provides the medical physicist group with a feedback system on the efficiency of daily QC tests, allowing them to understand the customized tolerance and maintain the machine. As shown in Figure 3, some red dots on the I‐chart of beam output change that exceeds the lower tolerance and action limits, which indicates the appearance of a system deviation. The physicists first repeated the QC test. If the QC data still exceeded the tolerance and action limits, it would find out if the cause could be resolved on its own. If not, it would be reported to the maintenance engineer for repair. Similarly, the red dots appeared on the I‐chart of isocenter KV offset and jaw X1, which reminded the physicist to take steps to improve the process. All data on the I‐chart of rotation‐induced couch shift and gantry relative exceed the lower tolerance limit but are within the lower action limit, which indicates that even though the process has a systemic error, it is manageable. The physicists need to have monitored the process but need not take action, which can save time for the physicist.

## 4 DISCUSSION

Three aspects of SSM to determine the limits in this study are critical. First, the control limit of the I‐chart is used as the tolerance limit. Second, it used the process capability index to determine action limits. Third, unspecified action limits are individually determined where the process is under control as defined by the I‐chart.

The Cp reflects the state of fluctuation inherent in the process while the Pp reflects the long‐term total fluctuation of the process, which meets the quality requirements.^24^ The distance between the daily QC test and the target value can directly reflect the fluctuation of data to estimate the sizes of Cp and Pp. The Collimation Rotation offset test was farthest from the target value which had the lowest Cp and Pp. While the Couch Rtn was the closest, the Cp and Pp were the largest. The process capability index quantitatively calculates the action limit, which indicates whether the physicist needs to take action to maintain the Linac in its current state. The tolerance limit is the minimum absolute value of control limits in the I‐chart. The I‐chart can monitor whether the data state is under control, and the tolerance limit can directly reflect the state of the Linac. This change does not require immediate action regarding the maintenance of the machine; however, it reminds the physical engineer to pay attention to the machine's status. The action limit and tolerance limit can effectively reflect the state of the Linac during this period so that the physicist can take reasonable measures and not waste time.

If action limits are not specified by clinical requirements, one should use process data to set reasonable action limits.^7^, ^40^ The SSM described herein requires that those action limits are determined using process data only over a range where there is good evidence that the process is not changing (under control).^34^ The process performance is distinct from the requirements of the process. One should always verify the tolerance limits against the action limits. If the tolerance limits are not within the action limits, then the process is not capable of meeting the clinical requirements and, thus, needs to be either re‐commissioned or re‐engineered to meet these requirements.^6^ In the I‐chart shown in Figure 3, all data of rotation‐induced couch shift and gantry relatively exceed the lower tolerance limit but are within the lower action limit, indicating that the process, despite being off the center, is in control. The I‐chart of the beam output change exceeds the action limit, which indicates that the process needs to be improved. The I‐chart provides the medical physicist group with a feedback system on the efficiency of the daily QC tests in the control stage, allowing them to understand the limits and maintain the machine.

Recently, the SSM has had some successful applications in radiotherapy. It was implemented in a radiotherapy department, allowing experts to repeat the breast repositioning matching procedure.^26^ Liu et al. were to develop and implement an automated plan check tool using an SSM with the aim of improving safety and efficiency in external beam radiotherapy.^23^ Rah et al. also used the SSM to provide an effective patient QA by setting customized tolerances.^34^ The SSM can provide an iterative and robust workflow to improve the efficiency and quality of the process, enabling a safer radiotherapy process.

In this study, Cpm and A could be combined into a single constant that is used when setting action limits^24^; however, they were left as separate constants for the purpose of clarity in presenting a new process. For unspecified processes in which the tolerance limits are wider than the action limits set, the clinician needs to either improve the process or choose a larger value of A. For existing processes, there could be sufficient historical data that indicates an increase in the value of A will not result in a negative clinical impact. For a new process, some types of prospective risk analyses such as failure modes and effects analyses could be done in conjunction with an increase in the value of *A* to minimize the probability of a negative clinical impact as a result of widening the action limits.^24^, ^41^

However, if the range uncertainties are greater than the tolerance level set, random variations in the measurement will lead to unnecessary intervention, increased downtime of equipment, and inefficient use of staff time. This overdetermine in tolerance tends to suggest that the underlying process is overwhelmingly out of tolerance when this may not be justified.^17^ Consequently, through a structured systematic QC using DMAIC, it is possible to balance patient safety versus resources available. The statistical analysis is built on the imprecision and bias measurements, as those change like the QC frequency or the QC equipment, you need to update them as inputs. If a change in maintenance or instrumentation changes the imprecision, you should recalculate the model. As was shown in the AAPM TG 142 report,^1^ some same QC tests have different tolerance limits with different QC frequencies. If the QC frequency was changed on the same QC test, the SSM would be reused to determine the tolerance limit.

A potential situation of SSM proposed in this study is that one may do a poor setup of daily QC tests during the collection of QC data for unspecified processes, which leads to larger uncertainties and wider limits. Then, wider tolerance and action limits will make it easier to pass QC tests. While this is true, once process control is established, each institution needs at least to compare the width of its limits in a meaningful way.^34^ For retrospective process analyses, one should use the largest number of under control data to calculate the action limits. The more data used, the better the estimate of the action limits.

In this study, whether a process is under control is determined by the I‐chart. The effect of the new limit is not evaluated in terms of time and economics. Having a comparison could better explain the positive effect of the SSM in the improvement of the QC process. In routine practice, implementation of SSM can be accomplished by following five stages, that is, the define stage, the measure stage, the analyze stage, the improve stage, and the control stage. We intend to compare the improved limits of daily QC tests with the maintenance record in our next work and further study the positive effect of the new limits.

Last, the presentation of this study considered quality metrics with equally likely upper and lower values. The SSM was used to determine the process for setting limits, which will also work for processes that produce unidirectional data such as gamma pass rates for patient QA; however, more efficient process capability index parameters exist for non‐normal QC data types.^42^ This is out of the scope of the current work; thus, we have left it for future investigations.

## 5 CONCLUSION

The SSM is an excellent framework to use in individually determining the limits of daily QC tests. Consequently, through a structured systematic process using DMAIC, it is possible that it balances patient safety and quality versus resources available and strikes a good balance between prescriptiveness and flexibility. The process capability index is an important parameter that provides quantitative information about determining the limits of QC tests.

## AUTHOR CONTRIBUTIONS

Min Ma and Minghui Li: Study conception, design, data acquisition, and wrote the paper; Ke Zhang, Pan Ma, Zhihui Hu, and Hui Yan: Drafted the manuscript; Kuo Men and Jianrong Dai: Drafted the manuscript and supervised the study; and all authors revised it critically for important intellectual content. All the authors have given final approval.

## CONFLICT OF INTEREST STATEMENT

The authors declare no conflicts of interest.

## Data Availability

The data that support the findings of this study are available from the corresponding author upon reasonable request.
